# Trends in Natural Nutrients for Oxidative Stress and Cell Senescence

**DOI:** 10.1155/2021/7501424

**Published:** 2021-07-01

**Authors:** Navid Omidifar, Mohsen moghadami, Seyyed Mojtaba Mousavi, Seyyed Alireza Hashemi, Ahmad Gholami, Mansoureh Shokripour, Zahra Sohrabi

**Affiliations:** ^1^Clinical Education Research Center, Shiraz University of Medical Sciences, Shiraz, Iran; ^2^Department of Pathology, Medical School, Shiraz University of Medical Sciences, Shiraz, Iran; ^3^Health Policy Research Center, Health Institute, Shiraz University of Medical Sciences, Shiraz, Iran; ^4^Department Chemical Engineering, National Taiwan University of Science and Technology, Taiwan; ^5^Nanomaterials and Polymer Nanocomposites Laboratory, School of Engineering, University of British Columbia, Kelowna, BC, Canada V1V 1V7; ^6^Biotechnology Research Center, Shiraz University of Medical Sciences, Shiraz, Iran; ^7^Nutrition Research Center, Shiraz University of Medical Sciences, Shiraz, Iran; ^8^Department of Community Nutrition, School of Nutrition and Food Sciences, Shiraz University of Medical Sciences, Shiraz, Iran

## Abstract

Due to the increase in the aged population and increased life expectancy, the underlying mechanisms involved in the aging process and cell senescence and the ways for modulating these processes in age-related diseases become important. One of the main mechanisms involved in aging and cell senescence, especially in the diseases related to aging, is the oxidative stress process and the following inflammation. Hence, the effects of antioxidants are highlighted in the literature due to their beneficial impacts on inhibiting telomere shortening or DNA damage and other processes related to aging and cell senescence in age-related diseases. Dietary components, foods, and dietary patterns rich in antioxidants can modulate the aging process and delay the progression of some chronic diseases such as cardiovascular diseases, diabetes, and Alzheimer's disease. Foods high in polyphenols, vitamin C, or carotenoids, olive oil, seeds, nuts, legumes, dietary supplements such as CoQ10, and some other dietary factors are the most important nutritional sources that have high antioxidant contents which can positively affect cell senescence and disease progression. Plant dietary patterns including Mediterranean diets can also inhibit telomere shortening following oxidative damages, and this can delay cell aging and senescence in age-related diseases. Further, olive oil can inhibit protein aggregation in Alzheimer's disease. It can be concluded that nutrition can delay the process of cell senescence in age-related diseases via inhibiting oxidative and inflammatory pathways. However, more studies are needed to better clarify the underlying mechanisms of nutrition and dietary components on cell senescence, aging, and disease progression, especially those related to age.

## 1. Introduction

According to the growth in aged population and increased life expectancy in different countries [1], more attention is paid to the mechanisms of aging especially at the cellular level [2].

**Figure 1:**
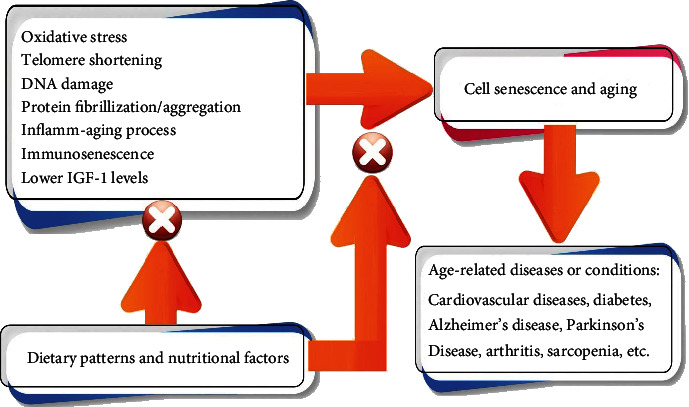
Factors affecting cell senescence and age-related diseases.

Among the theories of aging, the theory concerning the free radicals explains the underlying mechanism of aging process in age-related diseases including diabetes, osteoporosis, dementia, atherosclerosis, and cardiovascular diseases [3, 4]. Different factors can affect cell senescence and the progression of age-related diseases (Figure 1). Oxidative stress is one of the key factors involved in cell damage or injury [5, 6]. Endogenous or exogenous agents could induce tissue or organ damage via oxidative stress [7]. One of the important mechanisms related to aging is about lower immunity toward pathogens and infections which can be defined as immunosenescence [8]. One of the main reasons regarding immune deficiency and aging involves the oxidative pathways. At the time of high levels of oxidative stress, products of peroxidation or lipid membranes including malondialdehyde (MDA) can activate nuclear transcription factors that are all associated with cell senescence and longevity [9]. These nuclear transcription factors include tumor protein p53, nuclear factor kappa-light-chain-enhancer of activated B cells (NF-*κ*B), and transcriptional protein AP-1 [10].

One of the major epigenetic factors related to aging is considered to be oxidative stress, and it can also cause low-grade inflammation. This proinflammatory condition can increase the level of inflammatory cytokines and markers including interleukin-6 and tumor necrosis factor alpha (TNF-*α*), which can all activate the NF-KB pathway and induce mitochondrial superoxide and oxygen reactive species (ROS) production [2, 10]. ROS accumulation can be damaging for various biologic molecules such as nucleic acids, proteins, and lipids which can result in mutations of nucleic acids, protein deactivation or damage, and peroxidation of lipids [7, 11–13] that can all be important in disease progression via cell senescence. Also, the DNA damage caused by ROS is closely related to the cellular senescence [14] that can be damaging in age-related diseases.

The process called inflamm-aging is an important basis for frailty, aging process, and cell senescence in humans especially in age-related diseases [15]. On the other hand, inflammation can induce oxidative stress in a vicious cycle [16] that can affect the aging process and disease progression.

The antioxidant defense system including the enzymes such as catalase, glutathione peroxidase (GSH), and superoxide dismutase (SOD) decreases substantially during the aging process and nutrition can regulate cell senescence and aging in the related diseases [17].

**Figure 2:**
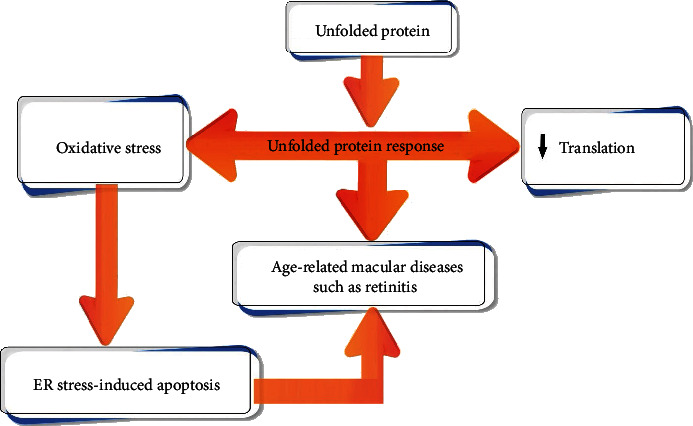
Unfolded protein response, oxidative stress, and age-related macular diseases. ER: endoplasmic reticulum.

Further, according to other mechanisms related to oxidative stress and age-related diseases, it can be mentioned that unfolded proteins in the endoplasmic reticulum (ER) can trigger unfolded protein response and this can in turn induce lower protein translation and higher levels of oxidative stress. The oxidative stress can cause ER stress-induced apoptosis and increase the risk of age-related macular diseases such as retinitis [18] (Figure 2).

## 2. Age-Related Diseases and Nutrition

It is demonstrated that nutrition is an important role modulator of aging process especially through the inflamm-aging process in age-related diseases [19–21]. Some dietary approaches or components were defined to affect aging in disease models [22].

**Figure 3:**
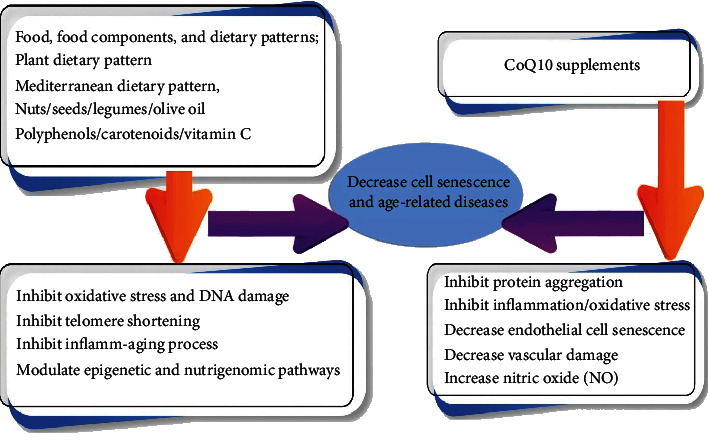
Dietary factors and their underlying mechanism affecting cell senescence and age-related diseases.

Effects of antioxidant nutrients on modulation of aging have been reported previously [23]. Other strategies affecting aging and cell senescence in diseases related to aging including cardiovascular diseases were also mentioned including calorie restriction without malnutrition [24], Mediterranean diet with olive oil (OO), and the like [25]. Mostly, the aforementioned dietary components could affect the inflamm-aging process and modulate the oxidative pathways [22, 26]. Dietary factors or patterns related to cell senescence are described here in details (Figure 3).

## 3. Plant-Rich Dietary Patterns

From the theories of aging and cell senescence, one of them is related to the telomere shortening. There is a high correlation between oxidative stress and telomere shortening which can accelerate aging and increase the risk of diseases such as cancer and cardiovascular diseases. It is reported that foods high in antioxidants (mostly plant foods) have beneficial effects against telomere shortening via inhibiting the oxidative damages [27, 28]. Effects of various plant foods including walnut [29], seeds, legumes, nuts [27], and olive oil [22], or plant dietary components such as polyphenols [30] and dietary carotenoids [5, 27], or plant-based dietary patters such as the Mediterranean dietary pattern [27] on inhibiting telomere shortening and delaying cell aging and age-related diseases were reported. Plant dietary patterns including most edible plants can synergistically modulate various processes such as oxidative stress, inflammation, telomere activity, and DNA methylation that are all associated with telomere attrition [31]. One of the major reasons for telomere shortening is related to oxidative stress damages due to the high content of guanines (5′-TTAGGG-3′) in telomeric DNA repeats [32]. Hence, it seems that plant foods high in antioxidants, especially nuts and seeds, can protect telomeres from oxidative damages and shortening [27]. In a clinical trial in healthy older adults, consumption of walnut for two years showed preventive effects on telomere attrition compared to the control group [29].

## 4. Polyphenols

Evidence showed that foods high in polyphenols can affect the aging process and cause protection against some age-related diseases including cataract, atherosclerosis, Alzheimer's disease, hypertension, arthritis, and diabetes. From these polyphenols, resveratrol and pterostilbene that are found in grape and blueberries can demonstrate antiaging properties through various mechanisms. These mechanisms include inhibiting oxidative pathways and inflammation and modulating cell senescence and telomere attrition [30]. It is demonstrated that resveratrol, as a potent polyphenol with antioxidant properties, could possibly increase the regulatory protein AROS expression (active regulator of Sirt1) and HuR (Hu antigen R). On the other hand, it could decrease DBC1 (deleted in breast cancer 1) and p53. These changes can show the antiaging effects of resveratrol as an important polyphenol via Sirt1 induction [33]. Sirt1 can affect autophagy as one of the mechanisms related to longevity [34]. In a study about a model of Alzheimer's disease, in the SAMP8 (senescence-accelerated mouse prone 8) mice, ingestion of resveratrol supplements for long term (1 g/kg) showed beneficial effects by activating the AMPK pathway and Sirt1 and this could affect cell survival and longevity. Further, resveratrol neuroprotective effects were reported while assessing the hallmarks related to Alzheimer's disease as an age-related disease [35]. In a study in the animal model, ingestion of various doses of resveratrol (30 and 100 mg/kg/day) in mice with premature aging of the ovaries, the protective effects of resveratrol against aging were observed and it could improve stem cell renewal due to its antioxidant properties through the activation of Nrf2 [30].

## 5. Vitamin C

One of the antioxidant nutrients that can modulate the process of inflamm-aging is vitamin C [36, 37]. Any deficiency in vitamin C can affect the demethylation of DNA and histones. This deficiency is present in the aging process. Vitamin C can have beneficial effects on delaying the aging process and age-related diseases [38].

Ascorbic acid or vitamin C is related to various molecular mechanisms associated with aging. This vitamin can modulate the free radical theory and inflamm-aging process by scavenging the free radicals and intercepting immunosenescence. It can also affect cell senescence via modulating nutrigenomic and epigenetic pathways that can be so important for the prevention of age-related diseases such as Alzheimer's disease, insulin resistance, atherosclerosis, and neurodegeneration [39]. In the epigenetic changes of DNA and histones, there are some enzymes including dioxygenase Fe^2+^ and 2-oxoglutarate (2OG-dependent) that need vitamin C for their activity. Hence, vitamin C can affect the epigenome and especially those changes concerned with the age-related diseases through modulating these enzyme activities. One of the major determinants of genome stability is related to the methylation of DNA, and vitamin C availability can affect this process. This can show the effects of vitamin C on aging process through epigenetic pathways [40, 41].

In an in vivo model, it was proposed that vitamin C supplementation can positively affect the aging process and life expectancy and it can reverse the abnormalities related to aging in various organs or tissues including liver and fat mass and those related to genomic stability. In addition to the improvement of inflammatory status following vitamin C use, normalization of AKT kinase phosphorylation, NF-kappa B at the transcriptional level, protein kinase delta (PKC delta), hypoxia-inducible factor-1 alpha (HIF1-alpha), and peroxisome proliferator-activated receptor alpha (PPAR-alpha) were reported that are all related to aging [42].

## 6. Coenzyme Q10

Coenzyme Q10 (CoQ10) can potentially increase cyclic adenosine monophosphate (cAMP) in the cells, and it can also enhance the antioxidant capacity in the mitochondria through the activation of SIRT1 and PGC-1*α*. As a result, this can modulate the cell senescence in the vascular endothelial cells [43]. SIRT1 is defined as an essential deacetylase that can increase nitric oxide (NO), and this can inhibit endothelial senescence [44, 45]. Further, it was reported that in an animal model supplemented with CoQ10, the mice had a higher metabolic rate related to fat via inhibiting the signaling pathway of CaMKII-MEK1/2-ERK1/2 and increasing cAMP levels [46]. In vitro studies claimed that CoQ10 bears anti-inflammatory functions in addition to its antioxidant properties in endothelial cells and it can delay the process of senescence by affecting miR-146a expression [47]. The findings regarding the effects of CoQ10 on the inhibition of cell senescence mostly focus on the dietary supplements and especially in the endothelial cells which can be important for the prevention of age-related diseases related to vascular aging [48].

## 7. Olive Oil

Olive oil can induce DNA protection against damage due to its phenolic components. Other compounds including tyrosol, oleuropein aglycone, caffeic acid, and oleuropein could show beneficial effects of olive oil due to scavenging free radicals and modulating the oxidative pathways [49] and this can demonstrate the beneficial effects of olive oil on reducing the risk of cancer, cardiovascular diseases, Parkinson's disease, Alzheimer's disease, and other age-related diseases [22]. Olive oil components including oleuropein and oleuropein aglycone can inhibit fibrillization of a protein called Tau that is one of the events happening in Alzheimer's disease (AD) pathogenesis [50]. On the other hand, olive oil phenolic compounds can inhibit DNA damage through protecting APEX1, a repair gene for DNA that is so essential for decreasing the vulnerability to age-related diseases [51].

As it was mentioned, telomere shortening is so important in the aging process, especially in the development of age-related diseases [52]. On the other hand, the activity of telomerase can be negatively affected by inflammation and oxidative stress [53]. However, in the diets rich in monounsaturated fats (MUFAs) such as the Mediterranean diet containing olive oil, lower rates of telomere shortening were reported and this was closely correlated with the lower levels of ROS in the cells and lower rates of apoptosis due to the direct effects of olive oil [54]. In particular, the oleuropein content of olive can decrease oxidative stress, which can directly affect cell senescence [22]. Moreover, olive oil can increase the catalase content in cells during aging that can be protective against oxidative stress during cell senescence [22] and it can cause protection against disease progression.

**Figure 4:**
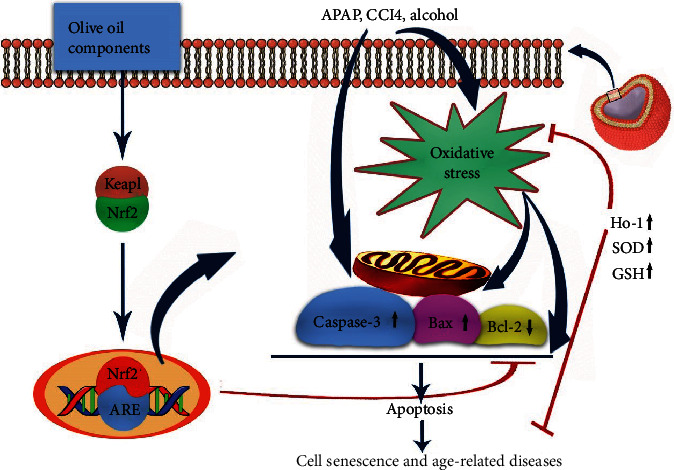
Olive oil effect on cell senescence and age-related diseases through the Nrf2 pathway.

Further, two components of olive oil named oleuropein and oleacein can also affect cell senescence [55]. These components can positively affect the cells, not only due to their antioxidant effects but also due to the stimulatory effects on the transcription factor Nrf2 and increased the expression of heme oxygenase-1 (HO-1). Nrf2 is an important transcription factor in the intracellular antioxidant defense system that can induce protection against cell apoptosis and cell senescence. HO-1 can also demonstrate antioxidant, anti-inflammatory, and antiapoptotic properties [56, 57] (Figure 4). All of the aforementioned effects can confirm the protective effects of olive oil against cell senescence via modulating oxidative stress [22].

Decreased levels of insulin-like growth factor-1 (IGF-1) were reported in age-related conditions such as sarcopenia [58, 59], diabetes, cardiovascular diseases, frailty, and the like [60, 61] (117, 121 of 4). Activation of the receptors for IGF-1 can upregulate the PI3K/AKT pathway, and this can promote cell survival and decrease cell senescence [62]. IGF-1 can beneficially affect the endothelial and cardiovascular system by increasing nitrite oxide (NO) availability, enhancing the antioxidant system, decreasing inflammation, decreasing cell death, and the like. Through these effects, IGF-1 can decrease the plaque size and the risk of cardiovascular diseases as one of the age-related diseases [63]. Research groups tried to affect aging through consumption of natural foods including olive oil which contains antioxidants that can modulate cell senescence and aging in age-related diseases or conditions [22]. It was reported that olive oil could possibly increase the levels of IGF-1 which can beneficially affect cell survival [64].

## 8. Conclusion

Because of the growth in aged population and age-related diseases including diabetes, arthritis, cardiovascular diseases, and Alzheimer's disease in various countries, modulating the aging process and cell senescence seems essential. Theory of free radicals and oxidative pathways for aging should be taken into account. Oxidative stress is highly intercorrelated with inflammation, and the process called inflamm-aging is an important basis for frailty, aging process, and cell senescence in humans, especially in the development of age-related diseases. It is obvious that dietary patterns and foods or food components or dietary supplements can modulate cell senescence via inhibiting oxidative stress, inflammation, or telomere shortening and DNA damage and prevent age-related diseases. This would be due to the phenolic compounds and antioxidants present in dietary components. From these nutrients or foods, foods including nuts, seeds, legumes, and olive oil and dietary components such as polyphenols, vitamin C, and carotenoids are of great importance to delay cell senescence in age-related diseases. Moreover, plant dietary patterns such as Mediterranean diet can positively affect telomere length or cell senescence and aging process and prevent the diseases related to aging. According to the dietary supplements, CoQ10 dietary supplements can also delay cell senescence via inhibiting oxidative stress and inflammation and inhibit vascular disease progression. However, more randomized clinical trials (RCTs) or in vitro studies are warranted to better elucidate the exact effects or mechanisms of action regarding the relationship between nutrition, oxidative stress, and cell senescence in age-related diseases.

## References

[1] Bloom D. E. (2011). 7 billion and counting. *Science*.

[2] Cannizzo E. S., Clement C. C., Sahu R., Follo C., Santambrogio L. (2011). Oxidative stress, inflamm-aging and immunosenescence. *Journal of Proteomics*.

[3] Vasto S., Candore G., Balistreri C. R. (2007). Inflammatory networks in ageing, age-related diseases and longevity. *Mechanisms of ageing and development*.

[4] Franceschi C., Campisi J. (2014). Chronic inflammation (inflammaging) and its potential contribution to age-associated diseases. *Journals of Gerontology Series A: Biomedical Sciences and Medical Sciences*.

[5] Gholami A., Ataei S., Ahmadimoghaddam D., Omidifar N., Nili-Ahmadabadi A. (2021). Pentoxifylline attenuates arsenic trioxide-induced cardiac oxidative damage in mice. *Oxidative Medicine and Cellular Longevity*.

[6] Gheitasi I., Azizi A., Omidifar N., Doustimotlagh A. H. (2020). Renoprotective effects of Origanum majorana methanolic L and carvacrol on ischemia/reperfusion-induced kidney injury in male rats. *Evidence-Based Complementary and Alternative Medicine*.

[7] Gholami A., Emadi F., Amini A., Shokripour M., Chashmpoosh M., Omidifar N. (2020). Functionalization of graphene oxide nanosheets can reduce their cytotoxicity to dental pulp stem cells. *Journal of Nanomaterials*.

[8] Cevenini E., Monti D., Franceschi C. (2013). Inflamm-ageing. *Current Opinion in Clinical Nutrition & Metabolic Care*.

[9] Gholami A., Mohammadi F., Ghasemi Y., Omidifar N., Ebrahiminezhad A. (2020). Antibacterial activity of SPIONs versus ferrous and ferric ions under aerobic and anaerobic conditions: a preliminary mechanism study. *IET nanobiotechnology*.

[10] Michaud M., Balardy L., Moulis G. (2013). Proinflammatory cytokines, aging, and age-related diseases. *Journal of the American Medical Directors Association*.

[11] Yuan Y., Hilliard G., Ferguson T., Millhorn D. E. (2003). Cobalt inhibits the interaction between hypoxia-inducible factor-*α* and von Hippel-Lindau protein by direct binding to hypoxia-inducible factor-*α*. *Journal of Biological Chemistry*.

[12] Wang X., Yokoi I., Liu J., Mori A. (1993). Cobalt(II) and Nickel(II) Ions as Promoters of Free Radicals _in Vivo:_ Detected Directly Using Electron Spin Resonance Spectrometry in Circulating Blood in Rats. *Archives of biochemistry and biophysics*.

[13] Sakagami T., Satoh K., Ishihara M. (2000). Effect of cobalt ion on radical intensity and cytotoxic activity of antioxidants. *Anticancer Research*.

[14] Muñoz-Espín D., Serrano M. (2014). Cellular senescence: from physiology to pathology. *Nature reviews Molecular cell biology*.

[15] Franceschi C., Bonafè M., Valensin S. (2000). Inflamm-aging: an evolutionary perspective on immunosenescence. *Annals of the new York Academy of Sciences*.

[16] Sohrabi Z., Eftekhari M. H., Eskandari M. H., Rezaianzadeh A., Sagheb M. M. (2016). Intradialytic oral protein supplementation and nutritional and inflammation outcomes in hemodialysis: a randomized controlled trial. *American Journal of Kidney Diseases*.

[17] vel Szic K. S., Declerck K., Vidaković M., vanden Berghe W. (2015). From inflammaging to healthy aging by dietary lifestyle choices: is epigenetics the key to personalized nutrition?. *Clinical Epigenetics*.

[18] Haeri M., Knox B. E. (2012). Endoplasmic reticulum stress and unfolded protein response pathways: potential for treating age-related retinal degeneration. *Journal of ophthalmic & vision research*.

[19] Santoro A., Pini E., Scurti M. (2014). Combating inflammaging through a Mediterranean whole diet approach: The NU-AGE project's conceptual framework and design. *Mechanisms of ageing and development*.

[20] Berendsen A., Santoro A., Pini E. (2013). A parallel randomized trial on the effect of a healthful diet on inflammageing and its consequences in European elderly people: design of the NU-AGE dietary intervention study. *Mechanisms of ageing and development*.

[21] Neufcourt L., Assmann K., Fezeu L. (2015). Prospective association between the dietary inflammatory index and metabolic syndrome: findings from the SU.VI.MAX study. *Nutrition, Metabolism and Cardiovascular Diseases*.

[22] Fernández del Río L., Gutiérrez-Casado E., Varela-López A., Villalba J. (2016). Olive oil and the hallmarks of aging. *Molecules*.

[23] Mitsui A., Hamuro J., Nakamura H. (2002). Overexpression of human thioredoxin in transgenic mice controls oxidative stress and life span. *Antioxidants and Redox Signaling*.

[24] Sohal R. S., Weindruch R. (1996). Oxidative stress, caloric restriction, and aging. *Science*.

[25] López-Miranda J., Pérez-Jiménez F., Ros E. (2010). Olive oil and health: summary of the II international conference on olive oil and health consensus report, Jaen and Cordoba (Spain) 2008. *Nutrition, metabolism and cardiovascular diseases*.

[26] Mousavi S. M., Hashemi S. A., Zarei M. (2020). Recent progress in chemical composition, production, and pharmaceutical effects of kombucha beverage: a complementary and alternative medicine. *Evidence-Based Complementary and Alternative Medicine*.

[27] Crous-Bou M., Molinuevo J.-L., Sala-Vila A. (2019). Plant-rich dietary patterns, plant foods and nutrients, and telomere length.. *Advances in Nutrition*.

[28] Omidifar N., Nili-Ahmadabadi A., Gholami A., Dastan D., Ahmadimoghaddam D., Nili-Ahmadabadi H. (2020). Biochemical and histological evidence on the protective effects of *allium hirtifolium boiss* (Persian shallot) as an herbal supplement in cadmium-induced hepatotoxicity. *Evidence-based complementary and alternative medicine*.

[29] Freitas-Simoes T.-M., Cofán M., Blasco M. A. (2018). Walnut consumption for two years and leukocyte telomere attrition in Mediterranean elders: results of a randomized controlled trial. *Nutrients*.

[30] Li Y. R., Li S., Lin C. C. (2018). Effect of resveratrol and pterostilbene on aging and longevity. *BioFactors*.

[31] Paul L. (2011). Diet, nutrition and telomere length. *The Journal of nutritional biochemistry*.

[32] Barnes R. P., Fouquerel E., Opresko P. L. (2019). The impact of oxidative DNA damage and stress on telomere homeostasis. *Mechanisms of ageing and development*.

[33] Li J., Zhang C.-X., Liu Y.-M., Chen K.-L., Chen G. (2017). A comparative study of anti-aging properties and mechanism: resveratrol and caloric restriction. *Oncotarget*.

[34] Morselli E., Maiuri M., Markaki M. (2010). Caloric restriction and resveratrol promote longevity through the Sirtuin-1-dependent induction of autophagy. *Cell death & disease*.

[35] Porquet D., Casadesús G., Bayod S. (2013). Dietary resveratrol prevents Alzheimer’s markers and increases life span in SAMP8. *Age*.

[36] Padayatty S. J., Katz A., Wang Y. (2003). Vitamin C as an antioxidant: evaluation of its role in disease prevention. *Journal of the American college of Nutrition*.

[37] Naidu K. A. (2003). Vitamin C in human health and disease is still a mystery? An overview. *Nutrition journal*.

[38] Cahill L. E., El-Sohemy A. (2009). Vitamin C transporter gene polymorphisms, dietary vitamin C and serum ascorbic acid. *Lifestyle Genomics*.

[39] Monacelli F., Acquarone E., Giannotti C., Borghi R., Nencioni A. (2017). Vitamin C, aging and Alzheimer’s disease. *Nutrients*.

[40] Young J. I., Züchner S., Wang G. (2015). Regulation of the epigenome by vitamin C. *Annual review of nutrition*.

[41] Yin R., Mao S.-Q., Zhao B. (2013). Ascorbic acid enhances Tet-mediated 5-methylcytosine oxidation and promotes DNA demethylation in mammals. *Journal of the American Chemical Society*.

[42] Chen B.-Y., Wang X., Chen L.-W., Luo Z.-J. (2012). Molecular targeting regulation of proliferation and differentiation of the bone marrow-derived mesenchymal stem cells or mesenchymal stromal cells. *Current drug targets*.

[43] Tian G., Sawashita J., Kubo H. (2014). Ubiquinol-10 supplementation activates mitochondria functions to decelerate senescence in senescence-accelerated mice. *Antioxidants & Redox Signaling*.

[44] Zarzuelo M. J., López-Sepúlveda R., Sánchez M. (2013). SIRT1 inhibits NADPH oxidase activation and protects endothelial function in the rat aorta: implications for vascular aging. *Biochemical Pharmacology*.

[45] Arunachalam G., Yao H., Sundar I. K., Caito S., Rahman I. (2010). SIRT1 regulates oxidant- and cigarette smoke-induced eNOS acetylation in endothelial cells: role of resveratrol. *Biochemical and biophysical research communications*.

[46] Xu Z., Huo J., Ding X. (2017). Coenzyme Q10 improves lipid metabolism and ameliorates obesity by regulating CaMKII-mediated PDE4 inhibition. *Scientific Reports*.

[47] Olivieri F., Lazzarini R., Babini L. (2013). Anti-inflammatory effect of ubiquinol-10 on young and senescent endothelial cells via miR-146a modulation. *Free Radical Biology and Medicine*.

[48] Huo J., Xu Z., Hosoe K. (2018). Coenzyme Q10 prevents senescence and dysfunction caused by oxidative stress in vascular endothelial cells. *Oxidative medicine and cellular longevity*.

[49] Fabiani R., Rosignoli P., de Bartolomeo A. (2008). Oxidative DNA damage is prevented by extracts of olive oil, hydroxytyrosol, and other olive phenolic compounds in human blood mononuclear cells and HL60 cells. *The Journal of Nutrition*.

[50] Daccache A., Lion C., Sibille N. (2011). Oleuropein and derivatives from olives as Tau aggregation inhibitors. *Neurochemistry International*.

[51] Erol Ö., Arda N., Erdem G. (2012). Phenols of virgin olive oil protects nuclear DNA against oxidative damage in HeLa cells. *Food and chemical toxicology*.

[52] de Vos-Houben J. M., Ottenheim N. R., Kafatos A. (2012). Telomere length, oxidative stress, and antioxidant status in elderly men in Zutphen and Crete. *Mechanisms of ageing and development*.

[53] Boccardi V., Esposito A., Rizzo M. R., Marfella R., Barbieri M., Paolisso G. (2013). Mediterranean diet, telomere maintenance and health status among elderly. *PloS one*.

[54] Marin C., Delgado-Lista J., Ramirez R. (2012). Mediterranean diet reduces senescence-associated stress in endothelial cells. *Age*.

[55] Parzonko A., Czerwińska M. E., Kiss A. K., Naruszewicz M. (2013). Oleuropein and oleacein may restore biological functions of endothelial progenitor cells impaired by angiotensin II via activation of Nrf2/heme oxygenase-1 pathway. *Phytomedicine*.

[56] Levonen A.-L., Inkala M., Heikura T. (2007). Nrf 2 gene transfer induces antioxidant enzymes and suppresses smooth muscle cell growth in vitro and reduces oxidative stress in rabbit aorta in vivo. *Arteriosclerosis, thrombosis, and vascular biology*.

[57] Dulak J., Loboda A., Jozkowicz A. (2008). Effect of heme oxygenase-1 on vascular function and disease. *Current opinion in lipidology*.

[58] Nasimi N., Dabbaghmanesh M. H., Sohrabi Z. (2019). Nutritional status and body fat mass: determinants of sarcopenia in community- dwelling older adults. *Experimental gerontology*.

[59] Nasimi N., Sohrabi Z., Dabbaghmanesh M. H. (2021). A novel fortified dairy product and sarcopenia measures in sarcopenic older adults: a double-blind randomized controlled trial. *Journal of the American Medical Directors Association*.

[60] Barzilai N., Huffman D. M., Muzumdar R. H., Bartke A. (2012). The critical role of metabolic pathways in aging. *Diabetes*.

[61] Rincon M., Rudin E., Barzilai N. (2005). The insulin/IGF-1 signaling in mammals and its relevance to human longevity. *Experimental gerontology*.

[62] Blume-Jensen P., Hunter T. (2001). Oncogenic kinase signalling. *Nature*.

[63] Higashi Y., Quevedo H. C., Tiwari S. (2014). Interaction between insulin-like growth factor-1 and atherosclerosis and vascular aging. *Cardiovascular Issues in Endocrinology*.

[64] OliClinomel N4 Study Group, Jia Z. Y., Yang J. (2015). Safety and efficacy of an olive oil-based triple-chamber bag for parenteral nutrition: a prospective, randomized, multi-center clinical trial in China. *Nutrition journal*.

